# Design, Calibration, and Application of a Wide-Range Fiber Bragg Grating Strain Sensor

**DOI:** 10.3390/s25041192

**Published:** 2025-02-15

**Authors:** Gang Wang, Jiajian Wang, Jian Meng, Liang Ren, Xing Fu

**Affiliations:** 1 State Key Laboratory of Offshore and Coastal Engineering, Dalian University of Technology, Dalian 116024, China; wanggang@mail.dlut.edu.cn (G.W.); fuxing@dlut.edu.cn (X.F.); 2 China Petroleum Pipeline Engineering Co., Ltd., Langfang 065000, China; mengjian-kc@cnpc.com.cn

**Keywords:** wide-range fiber Bragg grating sensor, extra-large strain monitoring, desensitization mechanism, 3D printing technology

## Abstract

To address the issue of extra-large structural deformation or strain in infrastructures such as bridges, buildings, railroads, and pipelines during catastrophic events, this study proposes a wide-range fiber Bragg grating (FBG) strain sensor utilizing a snake spring desensitization mechanism to share large parts of the strains. Initially, the axial stiffness of the snake spring desensitization mechanism was derived using the strain energy method, which was applied for stiffness calculation, range determination, and parameter design of the entire structure, where the snake spring and the FBG strain sensor were connected in series. Then, the snake springs were fabricated using 3D printing technology and assembled with the FBG sensor to construct a wide-range strain sensor. The wide-range sensor was subsequently calibrated, achieving a strain range of 10,000 με and a linearity coefficient above 0.9995. Finally, the sensor was installed in a pipeline for testing, yielding favorable results. These results demonstrate that the proposed sensor exhibits a wide strain monitoring range and can be effectively used for real-time structural safety analysis by continuously monitoring localized large structure strains.

## 1. Introduction

Structural health monitoring, which refers to the use of in situ nondestructive sensing technology and structural system characteristics analysis to detect changes in structural properties and reveal structural damage or performance deterioration, has been suggested to be an important research direction in various engineering areas [1]. Initially, it was mainly applied in aerospace and mechanical engineering, and then was gradually expanded to civil engineering [2]. As the catastrophic failure of engineering structures is often accompanied by significant localized strains, strain serves as a key indicator in assessing structural safety conditions [3], such as the following: the dislocation of bridge decks and yielding of cables due to earthquakes or strong winds [4]; the progressive collapse of high-rise buildings caused by fire [5]; the flexural deformation of railroads, oil and gas pipelines, and other critical infrastructure as a result of landslides, geological fissures, and karst subsidence [6]; and the cracking of roadbeds and dams induced by uneven settlements [7]. During these catastrophic events, strain at critical stress points within the structure rises sharply, such as the tensile steel bar in the building, which reaches 7000 με in the earthquake. Therefore, the continuous monitoring of localized large strains can not only ensure the real-time management of structural safety, but also offer a scientific basis for identifying and understanding the damage and collapse of mechanisms [8,9,10].

Strains can be measured through different techniques. The most popular one is based on the change in electrical resistance during straining, and these sensors are called electric strain gauges (ESGs) [11]. ESGs offer advantages such as excellent linearity, compactness, and low cost. However, the strain range of ESGs is typically limited to approximately 2%. Although various methods have been proposed to expand both the static and dynamic measurement ranges of ESGs [12,13], the electric resistance change caused by strain is usually small. As a result, the transmission of these subtle resistance changes requires high-quality wiring, which significantly increases wire costs. At the same time, wires that are too long are susceptible to noise interference during the transmission of weak resistance changes, significantly reducing signal accuracy [14].

Fiber Bragg grating (FBG) sensors, a relatively new fiber-optic technique where structural strain influences the peak wavelength of the reflected wave from in the FBG, constitutes an inscribed Bragg grating and encapsulated protective packaging [11]. FBG sensors offer advantages such as small size, light weight, high accuracy, resistance to electromagnetic interference, corrosion resistance, low-temperature tolerance, and the ability to form large-scale quasi-distributed sensor networks [15]. Additionally, FBG sensors have been proven to be more robust than ESGs for the long-term monitoring of engineering structures [16,17,18,19]. Therefore, FBG sensors are currently the most widely used sensors in the field of structural health monitoring [20,21,22]. Generally, due to the inherent deformation capacity of quartz fiber, the strain range of FBG sensors made from silica fiber is limited to approximately 0.4–0.6%. Clearly, standard FBG strain sensors effectively meet the requirements of most tasks. However, their range may be insufficient for certain specialized tasks. Therefore, studies have been conducted to explore methods of extending the range of FBG strain sensors to achieve large-strain measurements in engineering structures. The measurement range of FBGs and distributed fibers can be extended in a simple and efficient manner using suitable taping methods [23,24]. However, this near-permanent installation complicates sensor replacement in the event of damage. These beam-based sensors were designed in [25,26], where the large strain of the overall structure was converted into the smaller strain of the FBG on the beam, enabling strain measurements exceeding 10%; however, the beam needs to be pushed directly or indirectly to bend it, which may affect the original structure’s stress field, thus limiting sensor compatibility. Ren et al. and Sun et al. [27,28] proposed an FBG strain sensor packaged by two gripper tubes. By adjusting the sensor’s support distance and fixing the packaging length of the bare FBG, an FBG strain sensor with a suitable strain sensitivity measurement range can be obtained.

Additionally, a series of FBG strain sensors with composite structures have been proposed to expand the strain measurement range, where components of differing stiffness are connected in series to form an integrated system. The stiffer element is designed to accommodate small deformation, while the more flexible component is intended for larger deformation. For instance, Zhou et al. [29] utilized a thermoplastic pipe to connect a spring and rustless steel slice with a fixed FBG, and the spring was used to share a large part of the strain resulting from the deformation of the structure. Furthermore, Zhu and Zhang et al. [30,31] developed a double-loop desensitization structure using TC4 or 304 stainless steel as the sensor substrate. Since the annular plane double-loop absorbs the majority of the deformation, the FBG is primarily employed to measure the residual, smaller strains. By decreasing the strain coupling coefficient between the FBG and the tested structure, a large-scale strain test was implemented. This approach requires machining metal to fabricate the sensing substrate. Compared to machining, 3D printing technology offers advantages such as complexity, mass production, customization, and low waste [32], and has been applied to the fabrication of various sensors, including seismometers [33], curvature sensors [34], and pressure sensors [35]. Therefore, integrating 3D printing with FBG sensing technology represents a promising direction for development.

In this paper, a wide-range FBG strain sensor based on a snake spring desensitization mechanism for ultra-large strain monitoring is proposed. Firstly, the factors affecting the measurement sensitivity and range of the sensor were analyzed based on the strain energy method, including the parameters of the FBG strain sensor and the snake spring structure. Then, based on high-precision, low-cost, and high-efficiency 3D printing technology, the snake spring desensitization mechanisms were processed and assembled with FBG strain sensors into a wide-range sensor. Finally, calibration and application tests on the sensor were carried out. The test results show that the measurement range of the composite sensor is greater than 10,000 με, demonstrating high linearity with a linearity coefficient above 0.9995, which can effectively solve the problem of large-strain measurement in structural health monitoring.

## 2. The Structure and Principle of the Wide-Range FBG Strain Sensor

### 2.1. Structural Design of the Sensor

**Figure 1:**
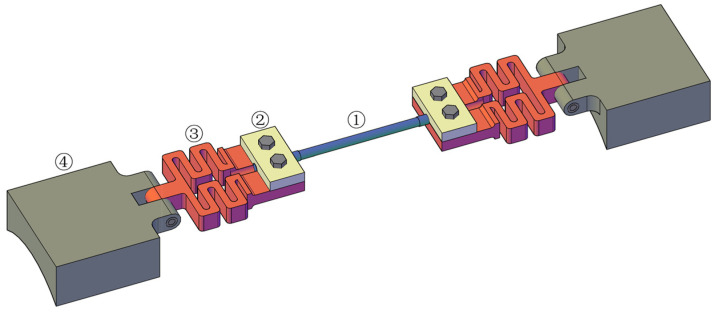
Three-dimensional diagram of the wide-range FBG strain sensor (① FBG strain sensor; ② fixed plate; ③ snake spring desensitization mechanism; ④ sensor support).

In this research, a wide-range FBG strain sensor was proposed, connecting a snake spring desensitization mechanism in series with the FBG strain sensor. And the snake spring was used to share a large part of the strain resulting from the deformation of the structure, and an FBG strain sensor was used to share the remaining small part of the strain. As depicted in Figure 1, the wide-range FBG strain sensor primarily consists of an FBG strain sensor, two snake spring desensitization mechanisms, and two sensor supports. The FBG strain sensor is encapsulated in a stainless steel tube, while the snake spring desensitization mechanism and sensor support were fabricated using 3D printing. Both ends of the FBG strain sensor were attached to the snake spring via fixed plates and bolts, while the opposite side of the snake spring was connected to the sensor support with pins. The sensor support can be affixed to the structure using an adhesive.

### 2.2. Theoretical Derivation of the Sensor

The range and sensitivity of the sensor are two important indexes. Thus, the relationship between the structural strain (*ε*) of the wide-range FBG strain sensor and the central wavelength shift (Δ*λ*_FBG_) of the FBG strain sensor was derived to determine the sensor’s measurement range and strain sensitivity.

**Figure 2:**
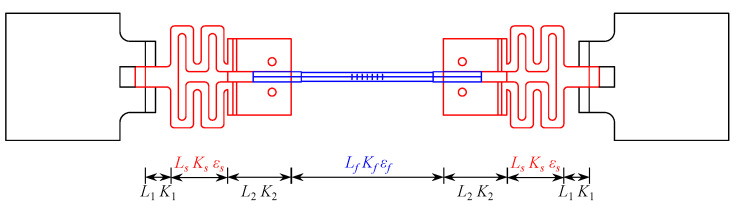
Principle of the wide-range FBG strain sensor.

The length, stiffness, and strain of each component of the wide-range FBG strain sensor are shown in Figure 2, and the distance of the sensor support is defined as *L* = *L_f_* + 2*L_s_* + 2*L*_1_ + 2*L*_2_. Assuming that the structural strain is concentrated primarily in the snake spring and FBG sensor, with respective deformations Δ*L_s_* and Δ*L_f_*, the structural deformation and strain can be represented as in Equation (1).

(1) $$\Delta L=2\Delta L_{s}+\Delta L_{f}$$

(2) $$\varepsilon =\frac{\Delta L}{L}=\frac{2\Delta L_{s}+\Delta L_{f}}{L}$$

According to the relationship between axial load, stiffness, and deformation, the following can be obtained:

(3) $$\begin{array}{l} F=K_{s}\Delta L_{s}\\ F=K_{f}\Delta L_{f} \end{array}$$

The deformation relationship between the snake spring and the FBG strain sensor is given by the following:

(4) $$\Delta L_{s}=\frac{K_{f}}{K_{s}}\Delta L_{f}$$

Substituting Equation (4) into Equation (2) yields the following:

(5) $$\varepsilon =\frac{2K_{f}/K_{s}+1}{L}\Delta L_{f}$$

The deformation of the FBG strain sensor is related to its length and strain.

(6) $$\Delta L_{f}=L_{f}\varepsilon_{f}$$

The relationship between wavelength shift and strain of FBG strain sensor is as follows:

(7) $$\Delta \lambda_{\mathrm{FBG}}=K_{\varepsilon }^{f}\varepsilon_{f}$$

Substituting Equations (6) and (7) into Equation (5) yields:

(8) $$\varepsilon =\frac{2K_{f}/K_{s}+1}{L}\frac{L_{f}}{K_{\varepsilon }^{f}}\Delta \lambda_{\mathrm{FBG}}$$

If the allowable wavelength shift range of the FBG strain sensor is [Δ*λ*_FBG_], the range of the wide-range FBG strain sensor can be obtained as follows:

(9) $$\left[\varepsilon \right]=\frac{2K_{f}/K_{s}+1}{L}\frac{L_{f}}{K_{\varepsilon }^{f}}\left[\Delta \lambda_{\mathrm{FBG}}\right]$$

The sensitivity of the wide-range FBG strain sensor is as follows:

(10) $$K_{\varepsilon }=\frac{L}{2K_{f}/K_{s}+1}\frac{K_{\varepsilon }^{f}}{L_{f}}$$

According to Equations (9) and (10), the main influential factors of the strain sensitivity of the wide-range FBG strain sensor include the sensitivity, stiffness, and length of the FBG strain sensor, as well as the stiffness and length of the snake spring. The permissible wavelength shift range [Δ*λ*_FBG_] of the FBG strain sensor is an additional parameter that significantly influences the range of the wide-range FBG strain sensor.

An FBG strain sensor packaged by two gripper tubes is employed as the core sensing element, where the bare FBG is encapsulated by a metal tube [27]. The fiber in both sides of the FBG is packaged with epoxy resin in the two gripper tubes, which are installed on the mounting supports by an adhesive or solder. Since the FBG area is not in contact with the epoxy resin, the FBG strain sensor eliminates the multi-peaks of reflective light from the FBG induced by the nonuniform bonding distribution of the epoxy resin. The parameters of this sensor are stable, and its performance is robust. Therefore, the stiffness and length of the snake spring structure will be analyzed next.

### 2.3. Stiffness Analysis of the Snake Spring

**Figure 3:**
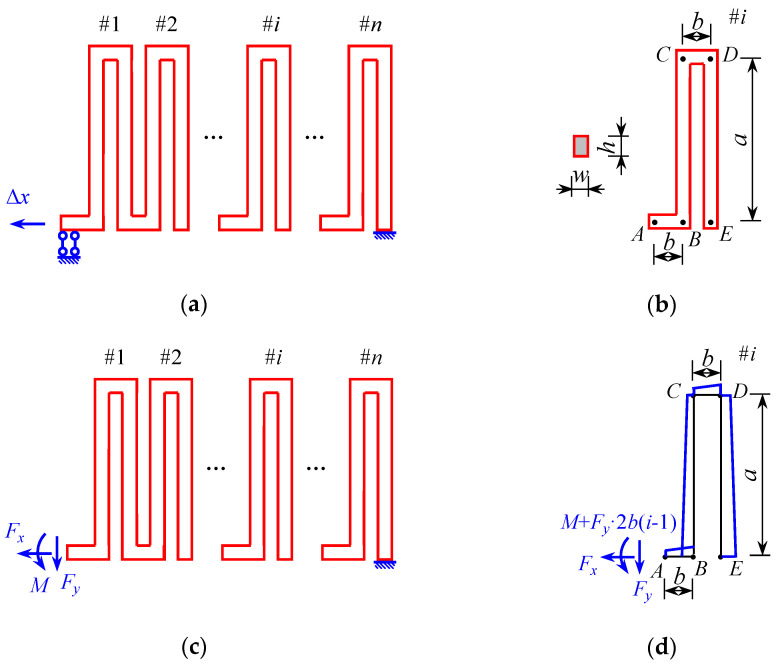
The structure of the unilateral snake spring: (**a**) boundary conditions of the unilateral snake spring; (**b**) the size of the snake spring element; (**c**) load analysis of the unilateral snake spring; and (**d**) moment diagram of the snake spring element.

The structural design of the sensor shows that most of the strain in the overall structure is primarily absorbed by the snake spring mechanism. The snake spring mechanism consists of two snake springs in parallel. A single snake spring is shown in Figure 3a, with boundary conditions simplified to fixed support at one end and directional support at the other. And the axial displacement acts on the directional support side.

Assuming that the length of each snake spring element shown in Figure 3b is *a* and the width is 2*b*, each snake spring element can be defined as the set of four beams: two primary beams of length *a* and two secondary beams of length *b*. Notably, the snake spring mechanism is typically designed with chamfered corners to reduce stress concentration, although this detail is disregarded in the theoretical analysis, which simplifies the structure to four complete beams. In the *i*-th snake spring element shown in Figure 3b, the main beams are *BC* and *DE*, and the secondary beams are *AB* and *CD*.

At the directional support, the loads are simplified into an axial force *F_x_*, a vertical force *F_y_* and a moment *M*, as shown in Figure 3c. In the *i*-th snake spring element shown in Figure 3d, the axial force is *F_x_*, the vertical force is *F_y_*, and the moment is *M* + *F_y_*·2*b*(*i* − 1), enabling the moment diagram to be plotted.

According to the load action of *F_x_*, *F_y_*, and *M* + *F_y_*·2*b*(*i* − 1), the internal force expression of the *i*-th snake spring element can be obtained as follows:

(11) $$\begin{array}{ll} M_{i}^{AB}=M+F_{y}\cdot 2b\left(i-1\right)+F_{y}\cdot x, & x\in \left(0,b\right)\\ M_{i}^{BC}=M+F_{y}\cdot b\left(2i-1\right)-F_{x}\cdot x, & x\in \left(0,a\right)\\ M_{i}^{CD}=M+F_{y}\cdot b\left(2i-1\right)-F_{x}\cdot a+F_{y}\cdot x, & x\in \left(0,b\right)\\ M_{i}^{DE}=M+F_{y}\cdot 2b\cdot i-F_{x}\cdot a+F_{x}\cdot x, & x\in \left(0,a\right) \end{array}$$

When the beam is bent, the strain energy is stored in the beam. During deformation, the bending strain energy of a beam equals the work carried out by external forces acting on it. Thus, the strain energy of the entire snake spring structure can be expressed as follows:

(12) $$\begin{array}{ll} U & =\sum_{i=1}^{n}\int_{0}^{l}\frac{M_{i}{\left(x\right)}^{2}}{2EI}dx\\ & =\sum_{i=1}^{n}\left(\int_{0}^{b}\frac{{\left(M_{i}^{AB}\right)}^{2}}{2EI}dx+\int_{0}^{a}\frac{{\left(M_{i}^{BC}\right)}^{2}}{2EI}dx+\int_{0}^{b}\frac{{\left(M_{i}^{CD}\right)}^{2}}{2EI}dx+\int_{0}^{a}\frac{{\left(M_{i}^{DE}\right)}^{2}}{2EI}dx\right) \end{array}$$

Because the partial derivative of the strain energy with respect to the load is the displacement in the corresponding directions, and the angle and vertical displacements are zero at the left-end support, it can be obtained as follows:

(13) $$\begin{array}{l} \frac{\partial U}{\partial M}=0\\ \frac{\partial U}{\partial F_{y}}=0 \end{array}$$

The relation of *F_y_*, *M* in respect to *F_x_* can be obtained according to Equation (13).

The relationship between load, displacement, stiffness, and strain energy is satisfied as follows:

(14) $$\Delta x=\frac{\partial U}{\partial F_{x}}$$

(15) $$k_{s}=\frac{F_{x}}{\Delta x}$$

The stiffness value *k_s_* of the unilateral snake spring can be obtained. Since the snake spring is a parallel structure, its stiffness can be obtained as follows:

(16) $$K_{s}=2k_{s}$$

It is assumed that the section of the snake spring is rectangular, the width and height are *w* and *h*, respectively, and the flexural stiffness of the section is the following:

(17) $$I=\frac{hw^{3}}{12}$$

It can be obtained that the stiffness of the whole snake spring structure is the following:

(18) $$K_{s}=\frac{Ehw^{3}\left(4a^{2}n^{2}-a^{2}+8abn^{2}+2ab+4b^{2}n^{2}\right)}{a^{2}n\left(4a^{3}n^{2}-a^{3}+20a^{2}bn^{2}-a^{2}b+28ab^{2}n^{2}-3ab^{2}+12b^{3}n^{2}-9b^{3}\right)}$$

The length of the snake spring is also an influencing factor for the sensor range and sensitivity, which meets the following requirements:

(19) $$L_{s}=2nb$$

**Figure 4:**
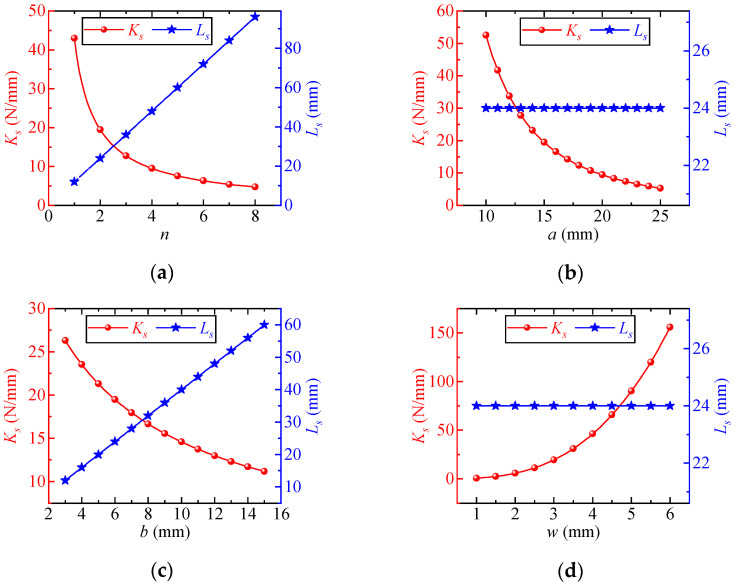
Influence of key parameters on the stiffness and length of snake spring: (**a**) element number *n*; (**b**) element length *a*; (**c**) element half width *b*; and (**d**) section width *w*.

According to Equations (18) and (19), Elasticity modulus *E* and section height ℎ are linear factors affecting the snake spring’s stiffness, without influencing its length. The influence degree of other parameters (element length *a*, half width *b*, element number *n*, and section width *w*) is analyzed through the control variable method. Figure 4 illustrates each parameter’s influence on the structural performance of the snake spring.

According to Figure 4 and Equation (18), the stiffness of the snake spring structure decreases as the element number *n*, the element length *a*, and the half width *b* increase, with varying influence degrees from each parameter. For *n* < 5 and *a* < 20 mm, the stiffness of the snake spring structure decreases significantly, gradually converging beyond these ranges. The stiffness decreases rapidly with an increase in half-width *b* initially and then gradually slowing. Additionally, increasing the section width *w* triples the stiffness of the snake spring structure.

As shown in Figure 4 and Equation (19), the snake spring length *L_s_* increases linearly with both the element number *n* and the half-width *b*, while element length *a* and section width *w* have no effect on *L_s_*.

## 3. Calibration Test of a Wide-Range FBG Strain Sensor

### 3.1. The Process of Calibration Test

**Figure 5:**
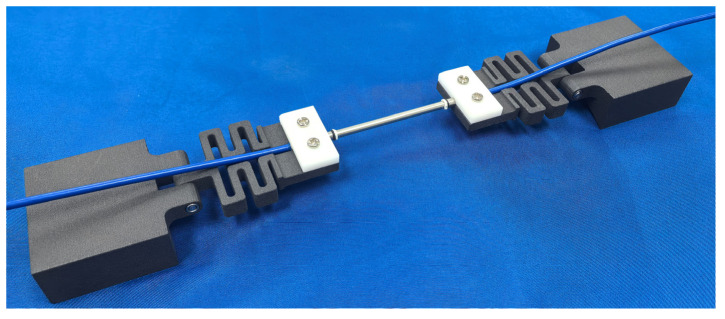
The prototype of the wide-range FBG sensor.

**Figure 6:**
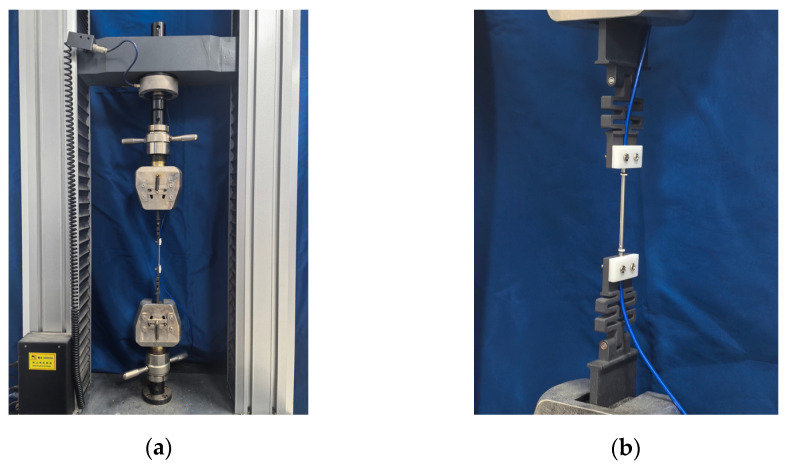
The calibration test: (**a**) testing machine and (**b**) a wide-range FBG strain sensor.

As shown in Figure 5, a wide-range FBG strain sensor was assembled using an FBG strain sensor, two snake spring desensitization mechanisms, and two sensor supports. And a snake spring and support were fabricated using 3D printing technology. The 3D printing material, named Future 7100 Nylon, is a gray-black polyamide 12 material characterized by high-temperature resistance, excellent toughness, and superior strength. It has a heat deflection temperature (ASTM D 648 @66 PSI [36]) of 129 °C and a flexural modulus (ASTM D790 [37]) of 1300 MPa, and is fabricated into a snake spring and support using selective laser sintering (SLS) technology. To calibrate the sensor performance with the testing machine shown in Figure 6a, the support at each end of the sensor is replaced with a thinner calibration plate to facilitate secure placement in the testing machine. Details are provided in Figure 6b.

In the calibration text, the sensor is initially secured in the testing machine and pre-stretched to eliminate assembly errors in individual connectors. The sensor is subsequently stretched to 1.75 mm, equivalent to a 10,000 με strain, before being unloaded back to 0 mm. This loading–unloading cycle is repeated ten times. Meanwhile, the FBG strain sensor’s wavelength data are recorded in real time.

### 3.2. Analysis of the Calibration Test

**Figure 7:**
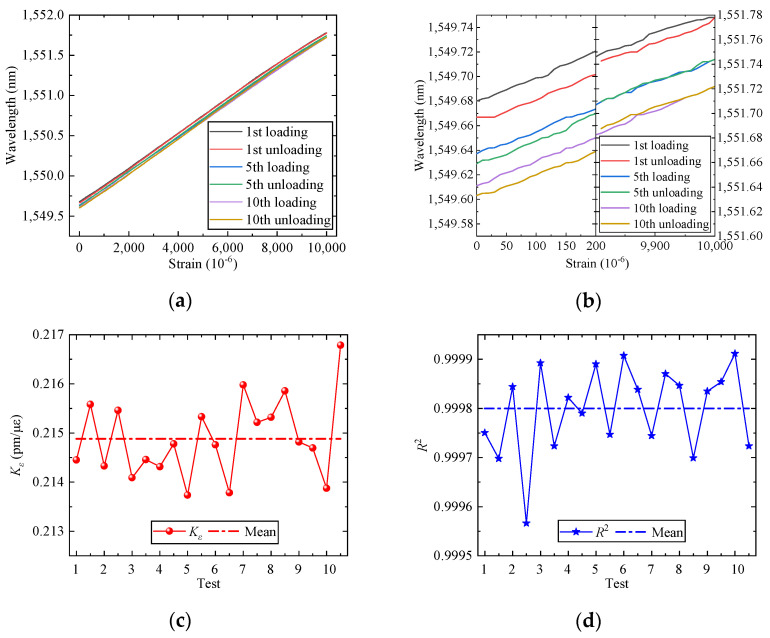
Calibration test results: (**a**) test data; (**b**) local test data; (**c**) sensitivity coefficients, and (**d**) linear coefficients.

The data for the first, fifth, and tenth times loading and unloading cycles is shown in Figure 7a,b. The high data consistency and a linear trend across cycles demonstrate that the wide-range FBG strain sensor has the advantage of good linearity and repeatability. The maximum hysteresis across ten tests is 1.152%, with an average of 0.87%, demonstrating that the sensor exhibits excellent hysteresis characteristics. Then, the data from each loading and unloading cycle were analyzed to derive sensitivity and linearity, as shown in Figure 7c,d. The sensor’s sensitivity ranged from 0.214 to 0.216 pm/με, with a linearity coefficient exceeding 0.9995, indicating high consistency and linearity. The various technologies can significantly enhance the range of FBG strain sensors, and the comparison of various wide-range FBG sensors is shown in Table 1.

From Table 1, there are significant differences in the ability of different technologies to enhance sensor performance, including strain range, sensitivity, and linearity, with the latter ideally being as close to 1 as possible. The application of different technologies results in FBG sensors with varying ranges, which should be selected based on specific application requirements and multiple standard tests.

## 4. Application Test of a Wide-Range FBG Strain Sensor

### 4.1. The Process of the Application Test

**Figure 8:**
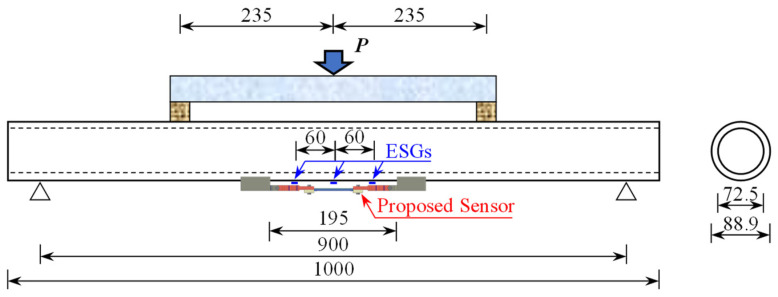
Schematic descriptions of the calibration test and loading conditions (unit: mm).

**Figure 9:**
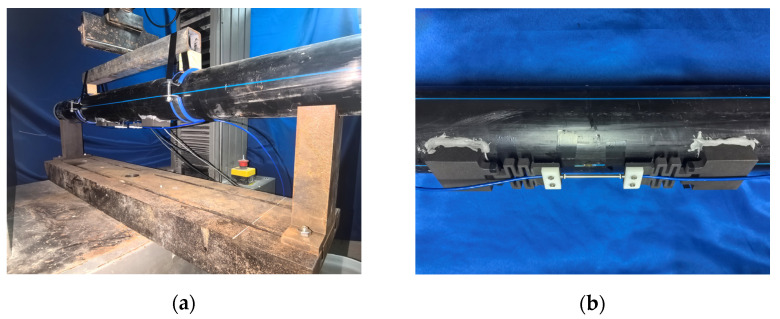
The application test: (**a**) testing machine and (**b**) a wide-range FBG strain sensor and ESG.

In this section, a wide-range FBG strain sensor was utilized for the surface strain monitoring of the high-density polyethylene (HDPE) pipeline tested in four-point bending during the entire loading process. The test specimen had a length of 1000 mm, an outer diameter of 88.9 mm, and an inner diameter of 72.5 mm. The length of the loading section is 470 mm. The wide-range strain sensor, spaced 195 mm between supports, and three strain gauges spaced 60 mm apart were attached to the lower part of the loading section. A concentrated displacement was applied in the mid-span of the distribution beam, and the displacement increased continuously from 0 mm to 22 mm at interval of 2 mm. Sample geometries, loading conditions, and sensor configuration are shown in Figure 8. The testing machine is presented in Figure 9a. As shown in Figure 9b, the wide-range FBG strain sensor and ESGs were bonded to the HDPE pipeline surface by utilizing high shear modulus glue with a shear strength of 22 MPa. The bending test was conducted in the laboratory under constant room temperature conditions. It could be noted that the range of strain is orders of magnitude higher than the effects of temperature changes. Therefore, temperature effects were disregarded.

### 4.2. Analysis of the Application Test

**Figure 10:**
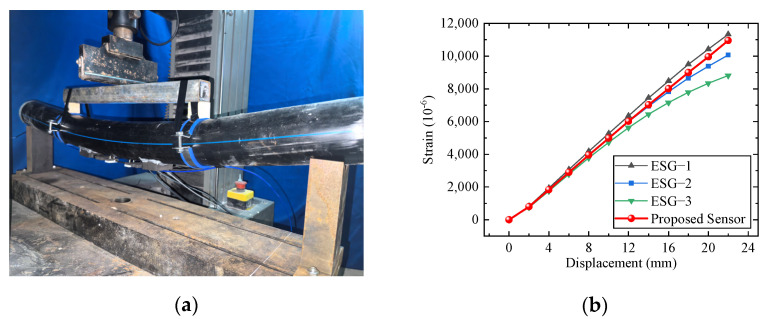
Application test results: (**a**) large deformation of the HDPE pipeline and (**b**) sensor data of the wide-range FBG strain sensor and ESG.

The results of large deformation of the HDPE pipeline are shown in Figure 10a. Due to the four-point bending load and the use of wooden blocks and collars at the loading points, local buckling is prevented, and the pipeline exhibits an overall large deformation. As shown in Figure 10b, the monitoring results of the three strain gauges are relatively close to one another, indicating that the loaded section of the pipeline functions as a pure bending section with uniform strain values at each point.

Additionally, the results of the wide-range strain sensor fall within the range of the three strain gauges’ measurements, as shown in Figure 10b, demonstrating that the proposed sensor effectively detects large strains in infrastructure structures while providing accurate measurements. This technology shows significant potential for applications in structural health monitoring of infrastructure.

## 5. Conclusions

This paper proposes a design method for the wide-range FBG strain sensor which can be used for extra-large strain monitoring in infrastructures. By connecting the snake spring desensitization mechanism in series with the FBG strain sensor, large structural strain is converted into a large strain in the snake spring and a smaller strain in the FBG, enabling wide-range strain monitoring. Based on the theoretical analysis, calibration test, and application test, the following conclusions can be drawn:

The main influential factor for the strain sensitivity of the entire wide-range FBG strain sensor include the sensitivity, stiffness, and length of the FBG strain sensor, as well as the stiffness and length of the snake spring. The permissible wavelength shift range [Δ*λ*_FBG_] of the FBG strain sensor is an additional parameter that significantly influences the range of the wide-range FBG strain sensor.

Structural stiffness of the snake spring decreases significantly with increases in the element number *n*, element length *a*, and half width *b*, and increases with greater section width *w*. Snake spring length *L_s_* grows linearly with *n* and *b*. *a*, *w*, and *h* have no impact on snake spring length *L_s_*.

The wide-range FBG strain sensor can monitor up to 10,000 με, with its sensitivity ranging from 0.214 to 0.216 pm/με and linearity coefficients exceeding 0.9995. The proposed sensor effectively senses oversized strains in infrastructures while delivering accurate measurements, making it highly suitable for structural health monitoring applications.

## Figures and Tables

**Table 1:** The comparison of various wide range FBG sensors.

Sensor Type	Measured Strain Range (με)	Linearity	Sensitivity (pm/με)	Test Number
Proposed sensor in [29]	12,000	0.9998	0.031	4
Proposed sensor in [30]	4000	/	/	/
Proposed sensor in [31]	500	0.9993	/	/
Proposed sensor in this paper	10,000	0.9998	0.215	10

## Data Availability

Data are contained within the article.
